# Improving obesity research: Unveiling metabolic pathways through a 3D In vitro model of adipocytes using 3T3-L1 cells

**DOI:** 10.1371/journal.pone.0303612

**Published:** 2024-05-31

**Authors:** Thayna Mendonca Avelino, Marta García-Arévalo Provencio, Luis Antonio Peroni, Romênia Ramos Domingues, Felipe Rafael Torres, Paulo Sergio Lopes de Oliveira, Adriana Franco Paes Leme, Ana Carolina Migliorini Figueira

**Affiliations:** 1 National Laboratory of Bioscience (LNBio), National Center of Research in Energy and Materials (CNPEM), Campinas, Brazil; 2 Department of Pharmacology Science, State University of Campinas (UNICAMP), Campinas, Brazil; Virgen Macarena University Hospital, School of Medicine, University of Seville, SPAIN

## Abstract

Obesity, a burgeoning global health crisis, has tripled in prevalence over the past 45 years, necessitating innovative research methodologies. Adipocytes, which are responsible for energy storage, play a central role in obesity. However, most studies in this field rely on animal models or adipocyte monolayer cell cultures, which are limited in their ability to fully mimic the complex physiology of a living organism, or pose challenges in terms of cost, time consumption, and ethical considerations. These limitations prompt a shift towards alternative methodologies. In response, here we show a 3D in vitro model utilizing the 3T3-L1 cell line, aimed at faithfully replicating the metabolic intricacies of adipocytes in vivo. Using a workable cell line (3T3-L1), we produced adipocyte spheroids and differentiated them in presence and absence of TNF-α. Through a meticulous proteomic analysis, we compared the molecular profile of our adipose spheroids with that of adipose tissue from lean and obese C57BL/6J mice. This comparison demonstrated the model’s efficacy in studying metabolic conditions, with TNF-α treated spheroids displaying a notable resemblance to obese white adipose tissue. Our findings underscore the model’s simplicity, reproducibility, and cost-effectiveness, positioning it as a robust tool for authentically mimicking in vitro metabolic features of real adipose tissue. Notably, our model encapsulates key aspects of obesity, including insulin resistance and an obesity profile. This innovative approach has the potential to significantly impact the discovery of novel therapeutic interventions for metabolic syndrome and obesity. By providing a nuanced understanding of metabolic conditions, our 3D model stands as a transformative contribution to in vitro research, offering a pathway for the development of small molecules and biologics targeting these pervasive health issues in humans.

## Introduction

Obesity has become a pandemic-level condition due to the increasing adoption of unhealthy lifestyles by the world population in recent decades. It is a leading cause of mortality in several countries, as it can lead to complications such as cardiovascular, diabetic, hepatic, and neoplastic diseases [1, 2]. Obesity is generally characterized by an excess of body fat resulting from a persistently positive energy balance [1].

Adipocytes play a central role in this scenario, as their ability to store excess calories in the form of triglycerides, which are packaged into large lipid droplets [3]. Recent research has shed light on their profound influence on other aspects of metabolic homeostasis, including a complex interplay with other metabolic processes in the body [4]. Because of the plethora of effects in which adipocytes act, its importance is firmly established, and due to its complexity, adipose tissue is considered essential and highly active metabolic organ, which communicates with nerve tissue, stromovascular cells and immune system, among other tissues and cells, receiving and exporting signaling molecules and responses to the whole organism [5, 6].

During the last 20 years, the functions of adipose tissue were expanded to include its dynamic function as an endocrine organ after the discovery of leptin [7], followed by adiponectin [4]. Nowadays, it is known that adipose tissue secretes many other proteins and signaling molecules, known as adipokines, that are responsible to regulate the systemic metabolic state, connecting diverse tissues and body functions [8]. Considering the adverse metabolic consequences of the disbalance on this tissue, the better understanding of its divergent functions has been emphasized and urged to lead to more rational therapy proposals, and, in this view, the proposals of new obesity models are a central piece [9].

Currently, there are two main types of obesity models available for research: cellular in vitro models and animal models. Animal models have the advantage of being able to mimic the physiological environment and connections between different organs and systemic responses in the body. However, they are costly, time-consuming, and may not always accurately represent the phylogenetic background of humans, limiting the translation of results to human populations. Additionally, the use of animal models raises ethical concerns related to the welfare of animals used for research purposes [10, 11], the principle of the 3Rs (Replacement, Reduction and Refinement) [10, 11] has been proposed to address some of these ethical concerns by promoting the development of alternative methods to minimize animal usage and suffering. While animal models remain an important tool for obesity research, alternative approaches such as in vitro models are being developed to complement or replace animal models where possible.

On the other side, most in vitro methods for studying obesity have been performed using monolayer cell culture, which is a well-established and cost-effective model for large-scale assays that requires less time to be performed. However, it is known that cells in monolayer culture are subject to vastly different microenvironmental and physical conditions than in vivo models [12]. Additionally, fundamental differences in cell behavior have been observed when comparing adipogenesis monolayer and 3D cell cultures. The effects of cell morphology and its 3D organization suggest that 3D cultures of adipocytes may improve the recapitulation of adipogenesis and metabolic function in the spheroid model, highlighting the need to develop and optimize these culture models to better mimic the in vivo microenvironment [9, 13].

In this context, here we developed and validated a model of study that involves the cultivation of adipocytes in a 3D cell culture system with an environment more closely of that one encountered in vivo, mimicking certain aspects of a healthy and obese adipose tissue. To achieve this goal, we applied a well stablished cell culturing system based on magnetic levitation [14, 15] to bioprinting the pre-adipocytes 3T3-L1 and differentiated them in adipospheres, which were treated with Tumor necrosis factor-alpha (TNF-α). As it is known, TNF-α is a multifunctional cytokine involved in many cellular and biological processes such as immune function, cell differentiation, apoptosis, and energy metabolism [16], also, it was reported that TNF-α can induce a state of insulin resistance in adipocytes [17]. In this way, we tested if the treatment with TNF-α generates a 3D culture in a pro-inflammatory environment, that could reproduce some metabolic similarities to an obese adipose tissue. To validate the efficacy of the developed adipospheres in terms of mimics the animal metabolic conditions, we compared their proteomic profiles with those of adipose tissue from healthy and obese mice (C57BL/6J). Our results provide a validated, feasible and translatable model to performing large-scale in vitro studies on obesity. Furthermore, our findings highlight some key pathways regulated in the adipose tissue under obese and insulin resistance conditions.

## Material and methods

### 3T3-L1 cell culture, spheroid assembly, and differentiation

3T3-L1 mouse preadipocytes were obtained from American Type Culture Collection (ATCC). 3T3-L1 mouse preadipocytes (passage 10–15) were cultured in high glucose (4.5 g/L) Dulbecco’s Modified Eagle Medium supplemented with 10% calf serum and 100 U/mL penicillin–100 mg/mL streptomycin at 5% CO_2_ and 37°C and harvested before reaching 70% confluence for spheroid assemble.

After reaching 70% of confluence, cells were counted using an automatic counter (Countess Thermo), following the manufacture instructions, and the nanomagnetic particles presented at NanoShuttle™ solution-PL (Greiner) was added in the proportion of 1uL for each 1x10^4^ cells, following the already reported methodology [14, 20].

For magnetization, cells were added to a 96-well culture plate (Greiner Bio-One 655970–96-well microplate, PS, well with F / chimney bottom, cell-repellent surface, clear, sterile), in the amount of 1,5x10^4^ cells/well. For spheroids formation, a magnetic drive with 96 magnetic cylinders (Greiner Bio-One 655830) was positioned below the culture plate, inducing cells to levitate and aggregate at the well centers to form spheroids. These spheroids were monitored via inverted microscopes, the cells remain for 24 hours incubated with magnets and kept in a humid incubator at 37° C and 5% CO_2_ for spheroid assemble.

The spheroids were differentiated into adipocytes though changes in the culture medium in two steps. First, through the induction cocktail composed of DMEM, 10% FBS, 1% w/v antibiotics (penicillin and streptomycin), 1μM dexamethasone, 1μg / mL of insulin and 0.5mM of 3-isobutyl-1-methylxanthine (IBMX), for 48h. After induction, the medium was aspirated and the maintenance medium, composed of DMEM, 10% FBS, 1% w/v antibiotics, 1 μg/mL insulin was added. The maintenance medium was changed every 48 hours until differentiation reaches 14 days. In the last 24h of differentiation, the spheroids were divided in two groups, a control (white adipospheres—WA) that received the maintenance medium, and the treated group (white adipospheres treated with TNFα—WA-TNF-α), that had the maintenance medium changed for DMEM supplemented with 0,5% BSA (Roche) and 2,5nM of tumor necrosis factor alpha (TNF-α) (Gibco).

### Cell viability and size measurement

To evaluate the viability state of spheroids, the adipospheres were harvested from the wells and placed individually in a white 96-well plate with 100 μL of culture medium. The ATP of each tested condition was quantified using a CellTiter-Glo® 3D Cell Viability Assay (Promega) following the manufacturer’s instructions. The luminescence was recorded in a Glow Max Plate Reader (Promega, USA).

In addition, the spheroids diameter of 8 samples was measured at day 1, 3, 5, 8 11 and 14 after assembling though bright field microscopy. For this, spheroids were manually transferred to white plates (Greiner Bio-One, 655075) and the images were captured with Operetta High Content Imaging System (Perkin Elmer, Waltham, MA, USA) and the diameter was quantified using Harmony Software (Perkin Elmer) [14].

### Animal model

Male C57BL/6J mice were purchased from Model Organisms Laboratory (LOM) mouse facility at LNBio/CNPEM (Campinas, SP, Brazil). Mice were weaned at 21 days old, being maintained on a photoperiod of 12:12 light/dark cycle, at 21–24°C, with free access to food and water and 3 animals per cage. They were randomly separated into two different groups: control group was fed a chow diet (Nuvilab CR1), and high fat diet (HFD) group was fed a 60% fat diet for 12 weeks (diet compositions are described in supplemental material; Pragsolucoes Biociencias, Jau—SP, Brazil). At the age of 17 weeks, mice were sacrificed using ketamine–xylazine (300mg/Kg-30mg/Kg) given intraperitoneally, followed cervical dislocation. The perigonadal white adipose tissue (WAT) and brown adipose tissue (BAT) were dissected and immediately frozen with liquid nitrogen. Later, they were stored at –80 C until use.

The Ethical Committee of CNPEM/LNBio “Comissão de Ética no Uso de Animais” (CEUA-CNPEM) (Supplementary Material) specifically reviewed and approved this study (approval identification 61). The protocols were done conformed to the guidelines for ethical conduct in the care and use of animals established by the Brazilian Society of Laboratory Animal Science (SBCAL/COBEA). Animals were treated humanely, to alleviate suffering.

### Microscopy

The lipid droplets and the nuclei were stained after spheroids were fixed for 1 hour in 4% formalin and washed 3 times in PBS. The LipidSpot (Biotium; cat. Number 70065-T) lipid marker probe and the DAPI (Biotium; cat. Number 40043) were added according to the manufacturer’s instructions. After staining with the fluorescent probes, the images were obtained using a TCS SP8 (Leica) confocal microscope.

### Gene expression

Spheroids were homogenized in 0,5 ml of TRIzol Reagent (Invitrogen) in order to extract the RNA according with manufacturing protocol. A minimum of 20 spheroids were used to make RNA for each condition, for this they were manually transferred to the tubes, lysed and their RNA were extracted. cDNA was synthesized using High-Capacity cDNA Reverse Transcription Kit (Invitrogen). Quantitative PCR reactions using SYBR-green master mix (Applied Biosystems) were run on Applied biosystem termocycler. After verifying its suitability, rpl27 and tbp were used to normalize gene expression. Fold changes were calculated using the 2−ΔΔCt method as described [18]. Primers used for the various genes are listed in S1 Table of S1 File.

### Glucose uptake assay

After spheroids formation and differentiation in WA, the spheroids were cleaned with PBS and they were maintained for 24 hours without insulin stimulation in complete DMEM high glucose (4.5g/L glucose). Immediately later, a serum starvation with DMEM low glucose (1g/L glucose) was done for 1 hour. The next step was stimulating the spheroids with or without insulin (100 nM) in DMEM high glucose, completed with 10% charcoal serum and 1% penicillin/streptomycin. After 30 minutes of stimulation, the medium was taken and the glucose content was quantified by colorimetric method (GOD-PAD; LABORLAB, SP, Brasil). The absorbance of the samples was measured by Clariostar (BGM LABTECH).

### IFN-y quantification—Indirect Sandwich enzyme-linked immunosorbent assays

IFN-y Sandwich ELISA assays were performed using Mouse IFN-y Uncoated ELISA Kit (Thermo Fisher, #88–7314) according manufacture instructions. Briefly, flat bottomed-96 wells plates (Immuno Maxisorp, Thermo Fisher) were coated with 100uL/well anti-mouse IFN-y antibody in 1X ELISA/ELISPOT buffer, and placed at 4°C, overnight. The wells were washed three times in phosphate buffer saline (PBS) containing 0.05% Tween 20 (PBS-T). To avoid nonspecific binding, the remaining microplate sites were blocked with 200 uL/well of ELISA/ELISPOT Diluent (1X) at room temperature for 1 hour. After washing, the wells were incubated with 100uL/well of negative control, standard curve (IFN-y diluted in 1X ELISA/ELISPOT buffer from 2,000 to 0 pg/ml) and samples (1:10 v/v) for 16h at 4°C. After washing three times with PBS-T, 100uL/well of biotinilated-detection antibody, anti-mouse IFN-y was added and incubated at room temperature for 1 hour. Further, Streptavidin-HRP conjugated was added 1:250, and incubated at room temperature for 30 minutes. Finally, after the same washing procedures, the reaction was determined by adding the TMB solution and incubated at room temperature for 15min. The ELISA reaction was stopped with STOP solution, and the absorbance was measured at 450 nm in an EnSpire Multimode Plate Reader (Perkin Elmer, USA).

### Proteomics

#### Proteolytic digestion

All samples were submitted to the same protocol of protein extraction and trypsin digestion as previously described [14]. Briefly, the samples were homogenized with lysis buffer (8M urea, 2M thiourea in 30mM Tris-HCl pH 8.5, containing 1mM EDTA, and 1mM PMSF). The proteins were quantified by Bradford method and an aliquot containing 10 μg of proteins was submitted to reduction with 5 mM dithiothreitol (DTT), for 25 minutes, at 56°C, and alkylated with 14 mM iodoacetamide (IAA), for 30 minutes, at room temperature, in the dark. The remaining IAA was removed by the addition of excess DTT. To reduce the final concentration of urea to 1 M, the mixtures were diluted with 50 mM ammonium bicarbonate buffer. Proteins were digested with trypsin (1:50, w/w), for 18 hours, at 37°C, and then, 1% formic acid (v/v) was added to stop the digestion. The tryptic peptides were desalted with C18 stage tips. To avoid bias during measurements, all data collection was randomized using the R (v3.4.0) environment.

#### LC-MS/MS analysis

The peptide mixture (2.0ml) was analyzed using an LTQ Orbitrap Velos (Thermo Fisher Scientific) mass spectrometer coupled to nanoflow liquid chromatography on an EASY-nLC system (Proxeon Biosystems) with a Proxeon nanoelectrospray ion source. Peptides were separated in a 2–35% acetonitrile gradient, in 0.1% formic acid using a PicoFrit analytical column (20 cm × ID 75,5 μm particle size, New Objective), at a flow rate of 300 nL/min, over 175 minutes, as previously described [19].

#### Proteomic data analysis

Raw data were processed using MaxQuant v1.5.8 software, and MS/MS spectra were searched against the Mus musculus UniProt database (released in December 2020, 63,724 sequences, and 28,586,808 residues) using the Andromeda search engine. A tolerance of 10 ppm was considered for precursor ions, and 1 Da for-fragment ions, with a maximum of two missed cleavages. A fixed modification of carbamidomethylation of cysteine and variable modifications of methionine oxidation and protein N-terminal acetylation were considered [14]. A 1% false discovery rate (FDR) was set for both protein and peptide identifications. Protein quantification was performed using the LFQ algorithm, with a minimal ratio count of 1 and a window of 2 minutes for matching between runs. Data were processed in Perseus v1.6.7.0 software, excluding reverse sequences and those identified “only by site” entries. Protein abundance was calculated based on the normalized spectrum intensity (LFQ intensity) and was log2-transformed. The significance was assessed using Student’s t-test (P-value < 0.05). Data visualization, pathway maps, and enrichment analysis was performed with fold-change (FC) and p-value threshold value of 2.0 and 0.05, respectively, using the software Metaboanalyst v 5.0 and MetaCore (Clarivate) [14, 20].

### Statistical analysis

The data were analyzed in GraphPad Prism software using t-student test nonparametric. Statistical significance was established at a p-value of less than 0.05. All data points were derived from three or more biological replicates. Replicates were defined through manufacturing of spheroids in different days, utilizing cells from distinct passage numbers to ensure comprehensive and diverse data representation.

### Ethical approval and informed consent

Experiments carried out with the animals were in strict accordance with the recommendations set forth in the Guide for the Care and Use of Laboratory Animals of the Brazilian National Council of Animal Experimentation (http://www.cobea.org.br/), the Federal Law 11.794 (October 8, 2008) and ARRIVE guidelines. The Institutional Committee for Animal Ethics of the Brazilian Center for Research in Energy and Materials (CEUA-CNPEM, License 61) approved all the procedures used in this study. The datasets used and/or analyzed during the current study are available from the corresponding author on reasonable request.

## Results

### The TNF-α treatment dramatically downregulates the genes involved in adipogenesis in adipospheres

The 3T3-L1 pre-adipocytes were cultured and assembled in spheroids using a previously stablished methodology [14, 15, 21]. Specifically, 1.5x10^4^ 3T3-L1 cells were bioprinted into spheroids and magnetized for 24h. Subsequently, the formation of a spherical and compact 3D cell structure was observed. Differentiation of 3T3-L1 spheroids to produce the adipose tissue spheroid model was performed by induction cocktail. In the last 24h of differentiation, the spheroids were treated with 2.5 nM of TNF-α [22], which is a cytokine associated with inflammatory processes, and that had been linked to the induction of insulin resistance in murine adipocytes by reducing the transcriptional activity of GLUT4 gene [23, 24]. Considering this, we investigated if the TNF-α treatment on fully differentiated 3T3-L1 spheroids was able to induce a pro-inflammatory environment that generating an insulin resistant adiposphere.

**Fig 1:**
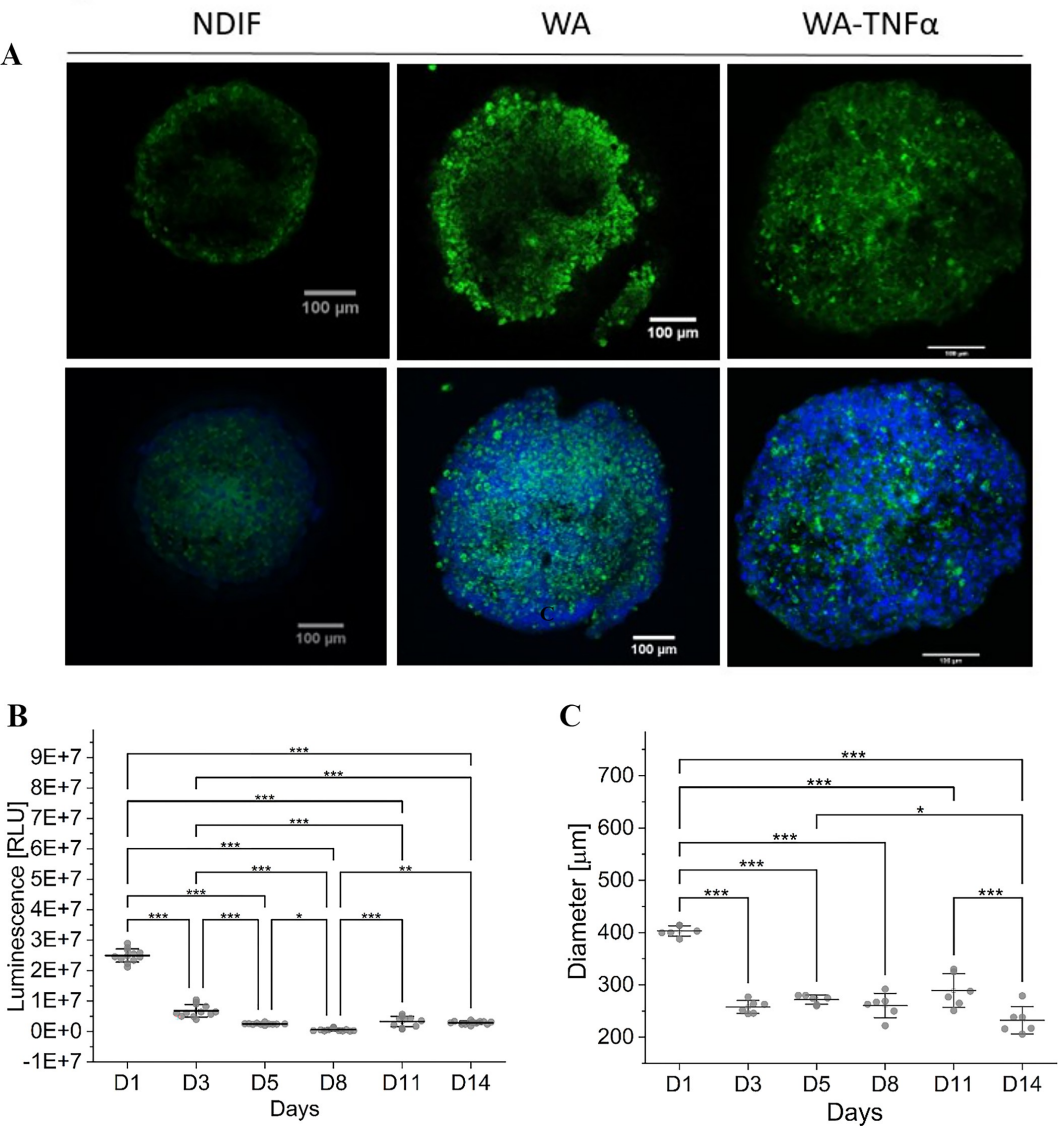
In panel, images from confocal scanning microscopy. Spheroid no differentiated in the first column (NDIF) and differentiated spheroids in subsequent columns (WA and WA-TNFα). The cell’s nucleus was stained with DAPI, lipid droplets were stained with LipidSpot and both staining were merged. Almost the whole space of adipocytes is occupied by lipid droplets after 14 days exposed to differentiated cocktail (WA and WA-TNFα) when compared with undifferentiated condition (NDIF). Confocal laser scan microscopy was applied to detect DAPI and LipidSpot. B) ATP quantification to analysis of spheroids viability through the time. C) Accompaniment of spheroid size through during the differentiation process. Statistical significance (One-Way ANOVA with Tukey post-test, where ***p<0,0005, **p<0,005 and *p<0,05). Error bars represent standard deviation of means from at least 7 replicates for size measurements and 12 replicates for viability.

Confocal scanning microscopy confirmed spheroid differentiation, through LipidSpot staining, assessing the increase in lipid content within the adipocyte cytoplasm. As shown in Fig 1A, differentiated spheroids (WA and WA-TNF-α) exhibited a greater accumulation of lipids compared to non-differentiated spheroids (NDIF). We observed that the TNF-α treatment did not influence the lipid accumulation, but promoted some rearrangement in cells organization on adipospheres compared with WA (Fig 1A), due to differences in fat labeling in both models, while WA presented a more peripheral labelling pattern, it seems to be more diffuse in WA-TNF-α.

The size and the viability of adipospheres were also measured during the 14 days of differentiation. The ATP production and diameter were checked at day 1, 3, 5, 8, 11 and 14 and the data obtained showed that until day 2 the culture seems to be in a period of high metabolic demand, probably because of the change of the type of culture from monolayer to 3D. From day 3, the spheroids reached stability on ATP production (Fig 1B), suggesting that cell viability was kept. Additionally, spheroid diameter (Fig 1C) was measured concomitantly to ATP quantification, exhibiting low variation until day 11, when the quantification indicated a reduction of the diameter, without cell death as founded in ATP quantification, suggesting that cell differentiation process is stabilized.

### Tumor necrosis factor-alpha induced insulin resistance in 3T3-L1 spheroids altering glucose uptake and cytokine secretion

**Fig 2:**
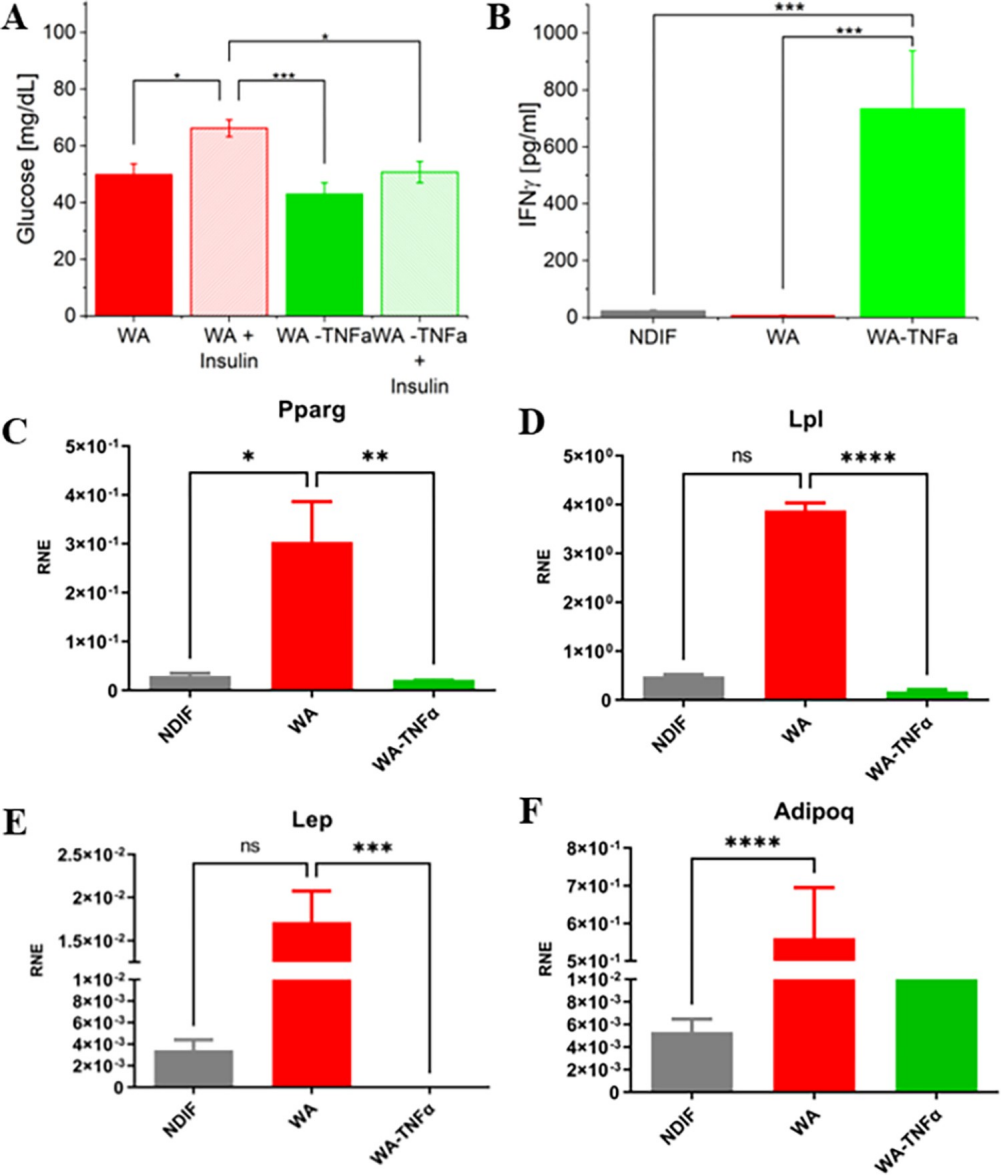
A) Glucose uptake assay of a metabolically healthy and insulin resistance in vitro model. B) Quantification of interleukin IFNγ secreted by the spheroids. Influence of differentiation coktail on the expression of adipocyte markers. Graphs display the mRNA expression profile, by qRT-PCR, from C) Pparg, D) Lpl, E) Lep and F) Adipoq, of differentiated 3T3-L1 spheroids (WA) and treated with TNF-α. Error bars represent standard deviation of means from at least 3 replicates; the measurement was performed after 14 days of differentiation.

To further investigate the central aspect of insulin resistance, we measured glucose uptake in both treated and non-treated spheroids in the presence and absence of insulin. Insulin-stimulated spheroids (WA-TNF-α + Insulin) showed significant difference in glucose uptake compared to non-treated spheroids (WA + Insulin), indicating that the TNF-α treatment effectively blocked glucose uptake and induced insulin resistance. In the control spheroids, without TNF-α treatment, the insulin-stimulated spheroids exhibited high glucose uptake levels in comparison to the group not exposed to insulin stimulation (WA). These results demonstrate the insulin sensitivity of WA adipospheres, with a transition to an insulin-resistant state observed following 24 hours of TNF-α treatment (Fig 2A). This validates our 3D adipose tissue model as representative of metabolic health in an obese individual and the manifestation of insulin resistance in obesity.

Another result that our WA-TNF-α spheroids mimic the metabolic physiology of an obese adipose tissue are related to proinflammatory cytokines quantification. Proinflammatory cytokines are overexpressed in obesity, leading to local and systemic inflammation, in which the IFN-γ levels in obese individuals correlate with BMI. IFN-γ is also elevated in obese animal adipose tissue and similarly, obese patients have higher levels of IFN-γ in serum and in visceral adipose tissue [25]. To understand the role of this cytokine in our adipospheres, we measure the IFN-γ secreted by cultures on day 14. At day 14 the TNF-α treated differentiated spheroids (WA-TNF-α) (Fig 2B) presented higher levels of secreted IFN-γ compared with the not treated spheroids (WA) and with the not differentiated ones (NDIF).

We further evaluate the effects of TNF-α treatment in the differentiation of pre-adipocytes into mature adipocytes. Through quantitative PCR analysis we measured relative mRNA expression of Adiponectin (Adipoq), Lipoprotein lipase (Lpl), Leptin (Lep), Peroxisome proliferator-activated receptor gamma (Pparγ), during adipogenesis. All the mRNA expressions were upregulated in WA spheroids in comparison to NDIF, indicating that adipocytes in spheroids achieved the expected differentiation, while the spheroids treated with TNF-α presented a downregulation in the same set of genes expression in comparison to the WA (Fig 2C–2F). These findings are in line with previous studies, which reported decreased gene expression related to adipogenesis in obese mice with type 2 diabetes mellitus [26].

### Tumor necrosis factor-alpha treated spheroids presents similarity to mouse adipose tissue in terms of proteome analysis

To provide further evidence of our WA and WA-TNF spheroids resemble the complex physiology of an animal model, the proteome of 3T3-L1 adipose spheroids was evaluated as an orthogonal method to compare the murine white adipose tissue from chow diet feeding mice (WAT), high-fat diet feeding mice (WAT-HDF), and the differentiated 3T3-L1-derived spheroids (WA and WA-TNF-α). Quantitative mass spectrometry (MS) and label-free protein quantitation (LFQ intensity) were used to analyze the protein content, and to compare their relative abundance in our different adipose models (WAT, WAT-HFD, WA, and WA-TNF-α), as previously described [14].

**Fig 3:**
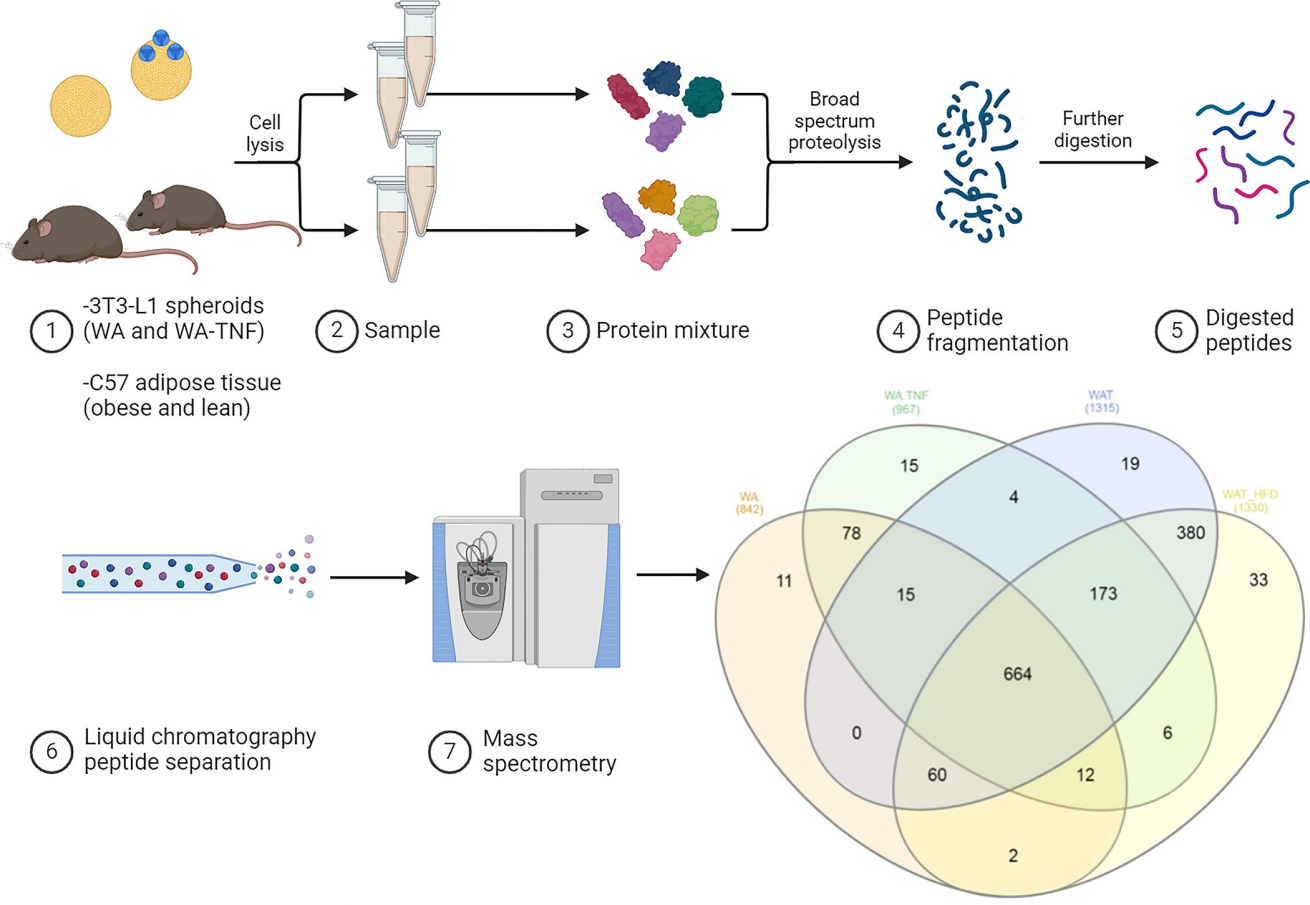
Pipeline of mass spectrometry-based analysis of adipose tissue from lean and obese mice (WAT and WAT-HDF) and from adipose tissue models (WA and WA-TNF); as a representative result, the Venn diagram of mass spectrometry-based proteomics of adipose spheroids (WA and WA-TNF-α) and white adipose tissue from mice (WAT and WAT-HDF). The samples were submitted to protein extraction; proteolytic digestion followed by MS analysis. Venn diagram of identified proteins, WA had 842 proteins identified, WA-TNF-α 967 proteins, WAT had 1315 and WAT-HFD 1330. WA has 11 exclusive proteins, WA-TNF-α has 15, WAT 19 and WAT-HDF 33 exclusive proteins identified.

The results show that 842 proteins were confidently identified for WA, while 967 proteins were identified for WA-TNF-α. For the WAT dataset, 1315 and 1330 proteins were quantified for WAT and WAT-HFD, respectively (Fig 3). The higher number of identified proteins in animal tissues was expected due to their higher complexity in comparison to 3D adipocyte cultures. Interestingly, 664 proteins were found to be common among all the analyzed groups. These amounts of common proteins represent 50–70% of the identified proteins and show similarities among the different adipose spheroids and animal models.

### Pathways enrichment analysis

To gain a better understanding of the signaling pathways involved in the differentially expressed proteins in the spheroids and mouse tissues, we used MetaboAnalyst 5.0 and Metacore™ software (Calrivate Analytics) to conduct enrichment analysis (EA), using a widely recognized protein-protein signaling database. The EA identified the protein IDs from the WA, WA-TNF-α, WAT, and WAT-HFD sets by the functional ontology function in MetaCore. The possibility of a random intersection of a gene set and the corresponding ontological entities was assessed using the hypergeometric intersection p-value. A lower p-value indicated that the object was more relevant to the dataset, suggesting a higher rating.

**Fig 4:**
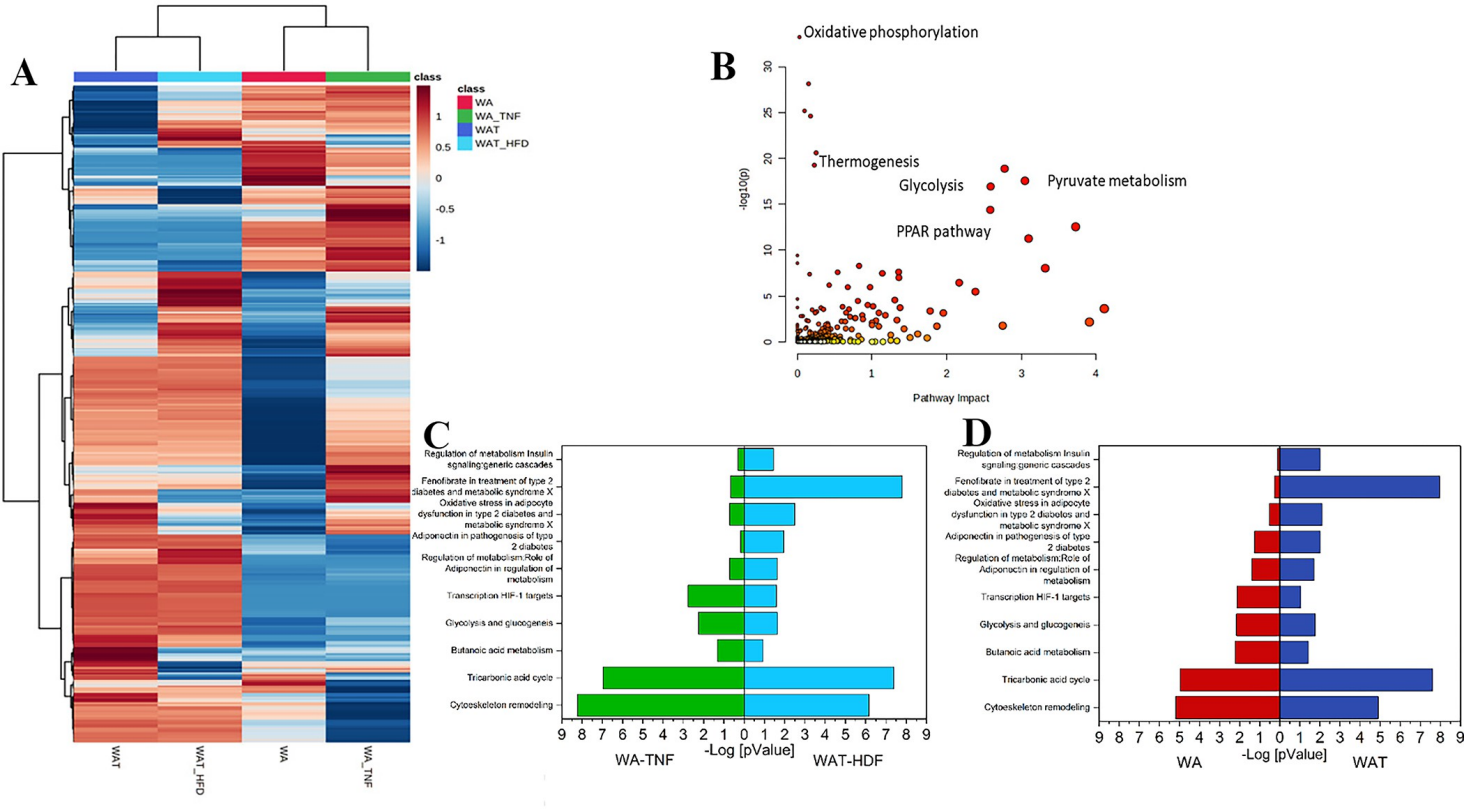
A) Heat map of mass spectrometry-based proteomics of adipose spheroids showing a comparative proteomic profile among adipose spheroids and adipose tissue from mice. B) Joint pathway analysis, the graph shows the pathways that are enriched. C) top 10 metabolic process networks; in red the process from WA and in dark blue from WAT. D) top 10 metabolic process networks; in green the process from WA-TNF-α and in dark blue from WAT-HDF.

To evaluate changes in proteomic profiles, biological replicates from WA and WAT were combined in the presence and absence of TNF-α (WA), or under chow or high-fat diets (WAT). The resulting heat map (Fig 4A) displays the differentially expressed proteins with a p-value of 0.05, revealing 600 significant differentially expressed proteins. Protein cluster analysis revealed that WAT and WAT-HFD are more similar to each other than to WA and WA-TNF-α, which is expected given that WAT is a tissue removed from animals and WA is a 3D cell culture from one cell type. However, some proteins displayed a closer relationship between the differentially expressed ones in WAT/WA and WAT-HFD/WA-TNF-α. The proteins from mitochondrial import inner membrane translocase subunit family (TIM 10 and 13) and Perilipin-2 (PLIN2) were found up regulated in WAT-HDF and WA-TNF-α, indicating an inflammatory and obesogenic environment [27, 28]. The overexpression of fatty-acid synthase (FAS) is obesity-related [29], our data show the expected up-regulation of this protein in HDF mice tissue but, a down regulation in WA and WA-TNF-α spheroids, this finding is related to the fact that the spheroids were not exposed to circulating fat as occurs in animal tissue. In general, our data shows that WA-TNF-α is more closely related to adipose tissue than WA.

Joint pathway analysis of the differentially expressed proteins in both evaluated models and tissues (Fig 4B) revealed the most impacted pathways (x-axis), which had a higher number of proteins differentially expressed in the comparison among all evaluated samples. Additionally, pathways with few proteins differentially expressed that may impact their correct function were represented by higher p-values (y-axis). Our analysis showed that changes in protein expression affected several metabolic pathways, particularly those related to thermogenesis, glycolysis, pyruvate metabolism, and the PPAR signaling pathway (Fig 4B). These findings present that our developed spheroid models reassemble important pathways associated with metabolic disorders, suggesting a very interesting toll for studies aiming modulation of thermogenesis, glycolysis, pyruvate metabolism, and the PPAR signaling pathways.

Fig 4C and 4D depict the enriched metabolic processes of WA x WAT and WA-TNF-α x WAT-HDF comparisons, respectively, revealing the top ten differentially expressed proteins that were upregulated in the adipose tissue of mice and spheroid samples. Insulin metabolism, fenofibrate action in type 2 diabetes (T2D), oxidative stress in T2D, adiponectin pathogenesis in T2D, regulation of metabolism and role of adiponectin, transcription of HIF1 targets, TCA cycle, and cytoskeleton remodeling were the common processes observed in all samples, indicating that the characteristic of adipose tissue is mimicked by differentiated adipospheres, demonstrating their ability to represent the adipose tissue.

Remarkably, the upregulation of several proteins associated with fatty acid metabolism and mitochondrial respiration, such as FABP5, TIM13, PLIN2, and PLIN5, was found in WAT-HFD and WA-TNF-α, whereas FABP4 and FAS were upregulated in WAT and WAT-HFD but downregulated in both spheroid models (Fig 4C and 4D). These results provide further evidence that our spheroid models recapitulate some of the important characteristics of real tissues, showing greater similarity to mouse tissue in our TNF-α-treated spheroid model.

**Fig 5:**
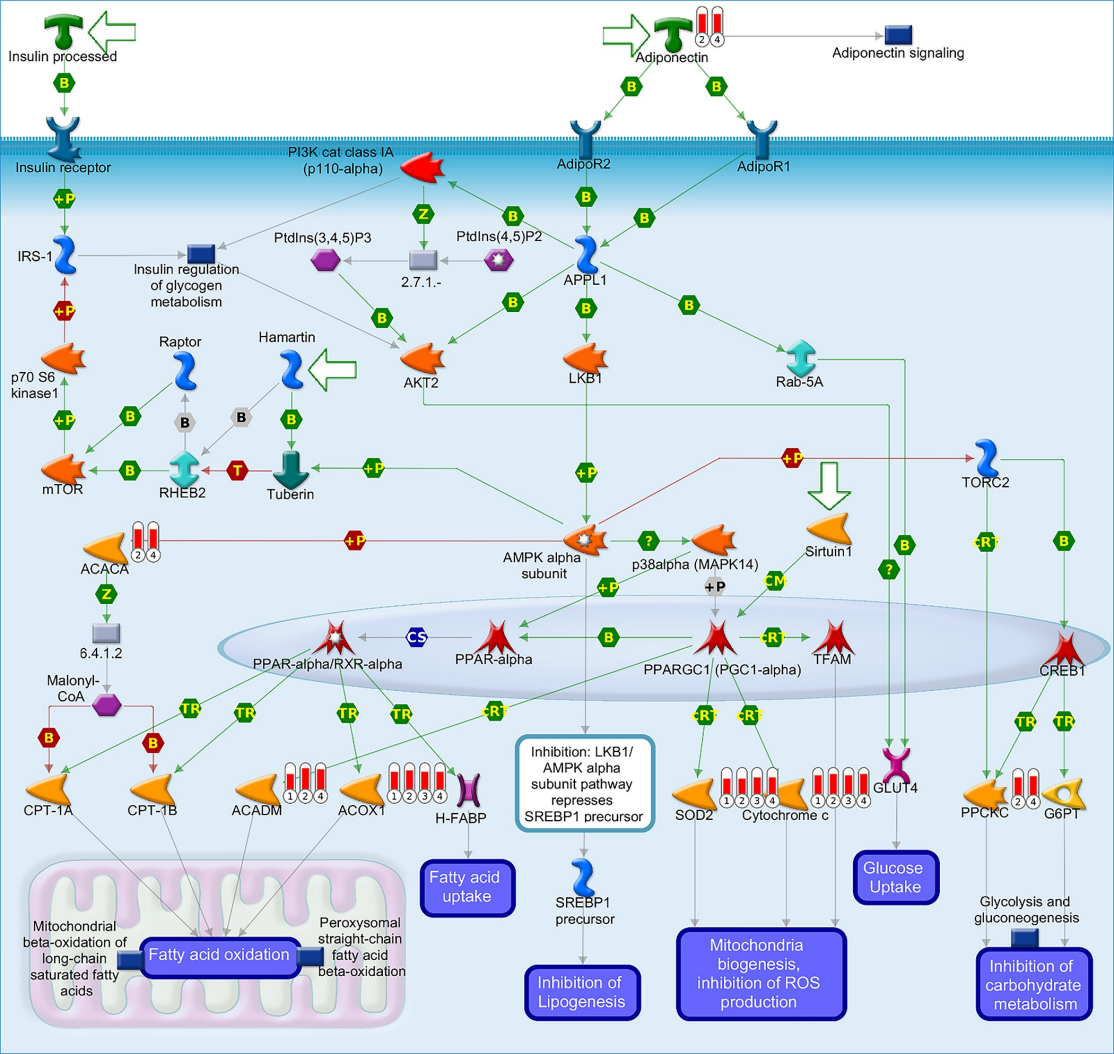
Regulation of metabolism, the role of adiponectin in regulation of metabolism. 1) WA, 2) WAT, 3) WA-TNF-α and 4) WAT-HDF. The pathway images were generated by GeneGo Metacore™ enrichment analysis. Well-characterized proteins or protein complexes are shown as individual symbols within the image; experimental data from all the records are connected and depicted as thermometer-like figures on the maps. Upward-facing thermometers are shown in red and indicate more abundant proteins. The linkage of proteins by arrows depicts the stimulatory and inhibitory effects or interaction of the encoded protein on the desired protein. Further explanations are provided at https://portal.genego.com/help/MC_legend.pdf.

**Fig 6:**
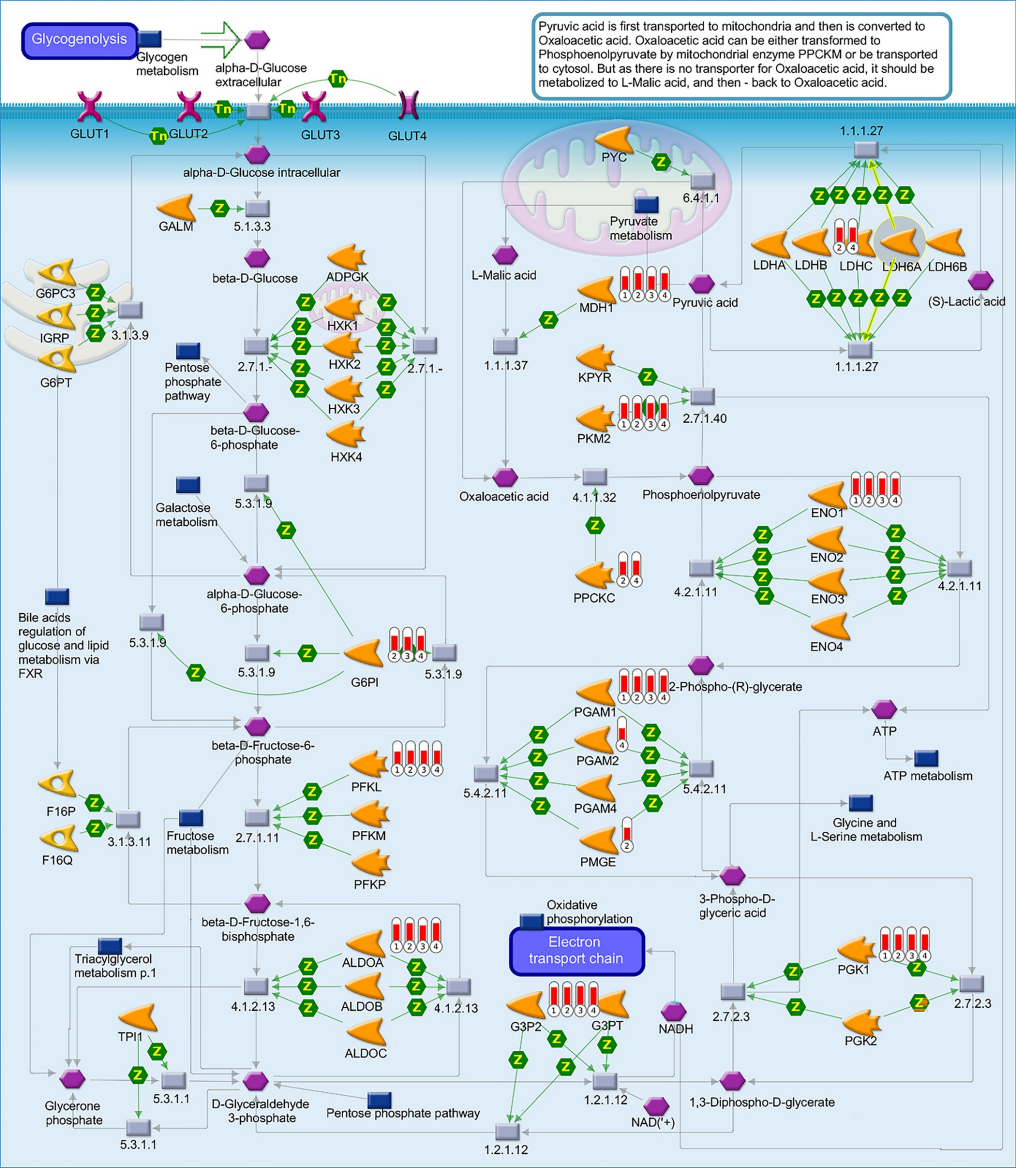
The map illustrates the process of glycogenolysis involving distinct components where 1) WA, 2) WAT, 3) WA-TNFα, and 4) WAT-HDF. These visual representations of pathways were created using GeneGo Metacore™ enrichment analysis. The image depicts well-defined proteins or protein complexes as separate symbols within the pathway. Experimental data from various records are represented as thermometer-like figures connected throughout the maps.

Additional evaluation in GeneGo Metacore™ enrichment analysis, which produced pathway maps were performed too further investigate the two most similar enriched metabolic processes in our data: (i) regulation of metabolism and the role of adiponectin in regulating metabolism, and (ii) glycogenolysis (Figs 5 and 6). The graphical representation of the pathway maps is based on the distribution of protein enrichment, in which well-characterized proteins or protein complexes are displayed as individual symbols, and the data collected from all experiments are shown and linked on the maps as thermometer-like symbols. An upward-facing red thermometer indicates upregulated proteins. In these results, we identified several critical hubs, including proteins involved in mitochondrial biogenesis, fatty acid uptake, and cell energy maintenance, all of which are regulated directly or indirectly by adiponectin (Fig 5). We found that among all the upregulated proteins, those involved in the adiponectin signaling pathway were most involved in mitochondrial biogenesis, inhibition of ROS production, and fatty acid oxidation, which are essential functions of adipose tissue. In Fig 6, we highlight the upregulated proteins that orchestrate the glycogenolysis process, with proteins involved in the electron transport chain, pyruvate metabolism, and ATP metabolism being the most upregulated proteins encountered in spheroids (WA and WA-TNF-α) proteome, similar to the results from mice adipose tissue (WAT and WAT-HDF).

## Discussion

Over the last 50 years, obesity has reached pandemic proportions worldwide, causing significant economic and societal consequences [30, 31]. This condition is characterized by an imbalance in energy expenditure and accumulation, which is associated with a persistent inflammatory condition often referred to as a sterile inflammatory process due to the absence of infectious agents.

Currently, obesity research relies heavily on animal models and two-dimensional cell culture models *in vitro*. However, animal models have ethical implications and limitations, and cell culture monolayers fail to represent the real structure and physiology of the tissues. Therefore, there is a growing demand for more refined models that can reproduce human and mouse white and brown/beige adipose tissues in vitro with physiological accuracy [30].

To establish an in vitro adipose tissue model reflecting physiological aspects, we generated spheroids from the widely used 3T3-L1 cell lineage in obesity research. These spheroids were then differentiated with or without TNF-α treatment, labeled as WA-TNF-α and WA, respectively. Importantly, the TNF-α-treated adipospheres (WA-TNF-α) distinctly exhibited insulin resistance. Notably, our data align with previous knowledge, indicating that TNF-α-treated adipocytes can replicate key transcriptional changes associated with insulin resistance and metabolic shifts [23, 31]. Emphasizing the significance, the integration of TNF-α treatment with the 3D culture approach proved crucial, rendering our model more relevant and robust in elucidating the functional outcomes of insulin resistance in vitro. The insulin resistance induced in the 3D models of mature 3T3-L1 cells (WA-TNF-α spheroids) has been first evidenced by changes in the expression of four adipocyte marker genes (Adipoq, Lep, Pparg, and Lpl), which were downregulated in WA-TNF-α spheroids compared to WA and NDIF. As previously reported, Lpl, which regulates adipose tissue, is significantly affected in insulin-resistant individuals, especially in the postprandial period [32]. Furthermore, PPARγ activation in mature adipocytes improves insulin sensitivity by inducing the expression of several genes involved in the insulin signaling cascade [33], and its downregulation is associated with insulin resistance. The role of leptin in the insulin metabolism remains unclear, with evidence suggesting that it can act as both insulin-sensitizing agent and inductor of insulin-resistant phenotype. In vitro observations suggest leptin plays an inhibitory role on glucose metabolism, while in vivo, it tends to play an insulin-sensitizing role due to central mechanisms [34]. Thus, our model of insulin-resistant spheroids by exposure to TNF-α for 24h reproduces the gene effects found in individuals resistant to this hormone.

To investigate the relationship between adiponectin secretion and the insulin resistance and adipocyte differentiation phenotypes in our in vitro obesity model, we measured adiponectin mRNA expression levels (Fig 2F). Our findings revealed that the adipokine adiponectin (Adipoq), had the mRNA expression decreased in WA-TNF-α. Adiponectin is known to promote adipocyte differentiation, insulin sensitivity, and lipid accumulation [35], and its high levels are associated with adipocyte differentiation, which tends to decrease with increasing adiposity in visceral adipose tissue [36]. On the other hand, low levels of adiponectin in circulation are linked to insulin resistance and obesity, which can alter glucose metabolism and insulin sensitivity [23, 37]. Therefore, our data show that both WA and WA-TNF-α adipospheres reached the expected differentiation stage for adipocytes, and WA-TNF-α downregulated at the end of differentiation, exhibiting an insulin-resistant phenotype.

In our assessment of the functionality of our adipospheres (WA and WA-TNF-α) through glucose uptake after insulin stimulation, our results showed that WA-TNF-α spheroids were not able to take up glucose like the non-treated ones (WA), even in the presence of insulin. These results agree with similar findings reported in stem cell-derived adipocyte models [38] provide further evidence that our TNF-α-treated adipospheres exhibit insulin resistance, whereas WA is still insulin sensitive. Therefore, our adiposphere models simulate adipose tissue from both metabolically healthy obese individuals and obese individuals with insulin resistance.

Additionally, we also observed increases in the inflammatory profile of our adipospheres after TNF-α treatment. The excessive presence of proinflammatory cytokines in obesity isn’t just linked to immune reactions within visceral adipose tissues (VAT), but also contributes to persistent systemic inflammation found in obesity. Elevated serum levels of IFN-γ in obese individuals showed significant associations with both overall obesity (measured by BMI) and central obesity (evaluated by waist-hip ratio) [39]. Similarly, higher levels of IFN-γ were found in adipose tissue from obese animals [40].

The most thorough validation of adiposphere models was performed by the comparison among them and with the real mouse tissue in a proteomic context. Proteomic studies of adipose tissue have previously been conducted to understand obesity and the regulation of gene expression, signaling, and metabolic changes in adipose tissue [41]. These studies have revealed gender-specific hallmarks related to redox status, immune responses, adipose tissue accumulation, and mitochondrial remodeling associated with aging and T2D [42]. In addition, comparison of subcutaneous and visceral adipose tissue identified novel protein species that may be involved in the obesity development in humans [43] and the analysis of distinct cellular components of adipose tissue shows that stroma vascular fraction, including pre-adipocytes, perivascular cells and blood cells are also important to secretion of adipokines and to response to regulatory signals [44].

Regarding the differences found in the number of identified proteins in our adipospheres models and in the mice tissues, which is explained by the complexity of the entire organ on face of a 3D culture composed by only one cell type, our data showed that proteins involved in the fatty acid and mitochondrial pathways are differently expressed in spheroids and in mouse adipose tissue. However, there is more similarity between WAT and WA model and WAT-HFD and WA-TNF -α model, suggesting two distinct models for evaluating adipose tissue, a lean and an insulin resistant one.

Our proteomic analysis provided valuable insights into protein interactions and cluster formations, allowing us to construct a directed network that elucidated crucial processes regulated in both adipose tissues and 3D models. These findings underscore the potential of these models in mimicking real tissues. Of note, among the top ten positively regulated metabolic processes, the role of adiponectin in metabolism was particularly noteworthy, especially for WAT-HDF and WA-TNF-α. Adiponectin, secreted by adipocytes, has been implicated in the development of insulin resistance [45]; our data showed that TNF-α-treated spheroids downregulated the relative mRNA expression of adiponectin and reduced the amount of secreted protein. Pathway analysis revealed that the pathway centrally regulated by adiponectin was enriched in our models.

Furthermore, our findings revealed that the translocase of the inner membrane complex (TIM13) was upregulated in both WAT-HDF and WA-TNF-α, consistent with previous studies that demonstrated its association with cardiac dysfunction in high-fat diet-induced obesity models [46]. We also observed upregulation of perilipin family proteins (PLIN 2 and PLIN 5) in both our WA-TNF-α and WAT-HDF, mirroring the expression pattern seen in obese mice. Perilipin proteins are known to be upregulated in obesity and have been linked to the regulation of lipid storage effects in obesity and insulin resistance-related non-alcoholic fatty liver disease [28, 47]. Taken together, our results show that our adiposphere models effectively recapitulate key molecular and functional changes observed in real adipose tissue, thus serving as a valuable tool for studying obesity-related metabolic disorders.

Our analysis yielded interesting findings indicating that the glycogen metabolism pathway is significantly regulated in both adipose tissue and adipose spheroids. We observed upregulation of several proteins (MDH1, PKM2, ENO1, TGAM1, PFKL, ALDOA, G3Q2), which play crucial roles in this metabolic pathway. This finding is consistent with previous reports suggesting that glycogen metabolism is critical in maintaining energy homeostasis, and that adipose tissue may contain glycogen stores [20, 48, 49]. Moreover, the accumulation of excess glycogen in adipose tissue has been proposed as a key feature of inflammatory-related metabolic stress in human obesity [48–50].

The 3D cellular models have emerged as highly effective tools in the development of innovative methodological approaches. However, in regulatory terms, there is a pressing need to compare Non-Animal Methods (NAMs) with animal models to compose a more comprehensive database. In this scenario, our finds provide a valuable tool to validate various types of NANs, this comparison is crucial, as NAMs have the potential to provide information of quality and relevance that rivals or even surpasses that obtained by traditional animal testing methods [51].

In summary, here we present a NAM with WA and WA-TNF-α spheroids models, which are suitable for investigating metabolic pathways associated with obesity, including insulin resistance, adiponectin regulation, and glycogenolysis. Additionally, our comparison of these models with adipose tissue from mice indicates that they accurately replicate key aspects of adipose tissue in vitro, including morphology, gene expression, and proteomic profile. Overall, our findings showed that these models are valuable tools for further studying the complex metabolic processes involved in obesity and related metabolic disorders.

## Conclusion

We have developed two 3D adipose tissue models, that closely mimic mouse adipose tissue, making them useful models for investigating metabolic pathways associated with obesity. Additionally, we also developed the TNF-α treated model, which mimics the phenotype and metabolic characteristics of an insulin resistant adipose tissue. These models replicate crucial aspects of adipose tissue, such as morphology, gene expression, and proteomic profile, indicating their high potential for studying obesity-related metabolic pathways. Through proteomic analysis, we identified key proteins involved in lipid storage regulation, insulin resistance, and obesity condition. Our findings demonstrate the capability of these models to mimic real tissue and enhance our understanding of the mechanisms underlying obesity-related metabolic disorders in special the insulin resistance and open an opportunity to be a feasible model to substitute the use of animals in the pre-clinical trials studies.

## Supporting information

S1 File (DOCX)

S2 File (ZIP)
