# Supplementary material for: Improving obesity research: Unveiling metabolic pathways through a 3D In vitro model of adipocytes using 3T3-L1 cells
Source: PLoS One. 2024 May 31;19(5):e0303612. doi: 10.1371/journal.pone.0303612 (PMC11142712; doi:10.1371/journal.pone.0303612)

Supplemental material

**Author names and affiliations:** Thayna Mendonca Avelino ^1,2^, Marta García-Arévalo Provencio ^1^, Luis Antonio Peroni ^1^, Romênia Ramos Domingues ^1^, Felipe Rafael Torres ^1^, Paulo Sergio Lopes de Oliveira ^1^, Adriana Franco Paes Leme ^1^, Ana Carolina Migliorini Figueira^1,2*^

1. National Center of Research in Energy and Materials (CNPEM), National Laboratory of Bioscience (LNBio)
2. State University of Campinas (UNICAMP) - Department of Pharmacology Science

**Corresponding author:** Ana Carolina Migliorini Figueira - [ana.figueira@lnbio.cnpem.br](mailto:ana.figueira@lnbio.cnpem.br)

Table S1 – Diet Composition

| Ingredient | Kcal (%) | Quantity g/ml |
| --- | --- | --- |
| Cornstarch | 0 | 0 |
| Casein | 19.8 | 258.5 |
| L-Cystine | 0.3 | 3.9 |
| Dextrinized cornstarch | 12.4 | 161.5 |
| Sucrose | 7.2 | 94.1 |
| Soybean oil | 5.6 | 32.3 |
| Lard | 54.6 | 316.6 |
| fiber | 0 | 64.6 |
| Mineral mix PSB10026B* | 0 | 64.6 |
| Vitamin mix AIN A 10X* | 0.1 | 1.3 |
| Choline chlorhydrate | 0 | 2.6 |

This diet provides 5217.3 kcal/kg; the composition in percentage of kcal is 20.1% protein, 19.7% carbohydrates and 60.2% fat. *For details, see Reeves et al. (Reeves, P. G., Nielsen, F. H., Fahey, G. C., Jr., AIN‐93 purified diets for laboratory rodents: final report of the American Institute of Nutrition ad hoc writing committee on the reformulation of the AIN‐76A rodent diet. J. Nutr. 1993, 123, 1939–1951.)

S2 - The Ethical Committee of CNPEM/LNBio


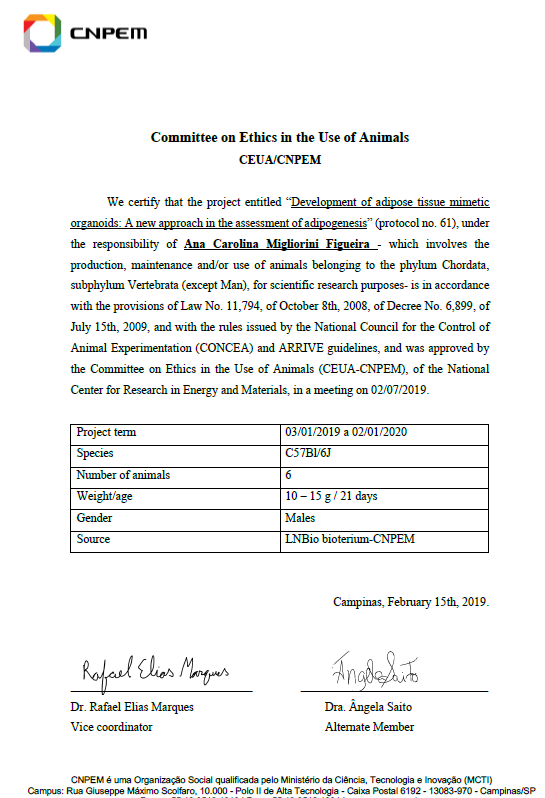

Supplement: S1 File — (DOCX) [file pone.0303612.s001.docx]
