# Supplementary material for: Improving obesity research: Unveiling metabolic pathways through a 3D In vitro model of adipocytes using 3T3-L1 cells
Source: PLoS One. 2024 May 31;19(5):e0303612. doi: 10.1371/journal.pone.0303612 (PMC11142712; doi:10.1371/journal.pone.0303612)
Supplement: S2 File — (ZIP) [file pone.0303612.s002.zip › Supl Material_Avelino PLOS/Sup Material_PLOS Avelino et al.pdf]

Supplemental material

Improving Obesity Research: Unveiling Metabolic Pathways Through a 3D In Vitro Model of Adipocytes using 3T3-L1 Cells

Author names and affiliations: Thayna Mendonca Avelino [1,2], Marta García-Arévalo Provencio [1], Luis Antonio Peroni [1], Romênia Ramos Domingues [1], Felipe Rafael Torres [1], Paulo Sergio Lopes de Oliveira [1], Adriana Franco Paes Leme [1], Ana Carolina Migliorini Figueira[1,2*]

1. National Center of Research in Energy and Materials (CNPEM), National Laboratory of Bioscience (LNBio)
2. State University of Campinas (UNICAMP) - Department of Pharmacology Science

Corresponding author: Ana Carolina Migliorini Figueira - ana.figueira@lnbio.cnpem.br

Table 1 – Diet Composition

Ingredient	Kcal (%)	Quantity g/ml
**Cornstarch**	0	0
**Casein**	19.8	258.5
**L-Cystine**	0.3	3.9
**Dextrinized cornstarch**	12.4	161.5
**Sucrose**	7.2	94.1
**Soybean oil**	5.6	32.3
**Lard**	54.6	316.6
**fiber**	0	64.6
**Mineral mix PSB10026B***	0	64.6
**Vitamin mix AIN A 10X***	0.1	1.3
**Choline chlorhydrate**	0	2.6

This diet provides 5217.3 kcal/kg; the composition in percentage of kcal is 20.1% protein, 19.7% carbohydrates and 60.2% fat. *For details, see Reeves et al. (Reeves, P. G., Nielsen, F. H., Fahey, G. C., Jr., AIN-93 purified diets for laboratory rodents: final report of the American Institute of Nutrition ad hoc writing committee on the reformulation of the AIN-76A rodent diet. J. Nutr. 1993, 123, 1939–1951.)

The Ethical Committee of CNPEM/LNBio

CNPEM

Committee on Ethics in the Use of Animals

CEUA/CNPEM

We certify that the project entitled "Development of adipose tissue mimetic organoids: A new approach in the assessment of adipogenesis" (protocol no. 61), under the responsibility of **Ana Carolina Migliorini Figueira** - which involves the production, maintenance and/or use of animals belonging to the phylum Chordata, subphylum Vertebrata (except Man), for scientific research purposes- is in accordance with the provisions of Law No. 11,794, of October 8th, 2008, of Decree No. 6,899, of July 15th, 2009, and with the rules issued by the National Council for the Control of Animal Experimentation (CONCEA) and ARRIVE guidelines, and was approved by the Committee on Ethics in the Use of Animals (CEUA-CNPEM), of the National Center for Research in Energy and Materials, in a meeting on 02/07/2019.

Project term	03/01/2019 a 02/01/2020
Species	C57Bl/6J
Number of animals	6
Weight/age	10 – 15 g / 21 days
Gender	Males
Source	LNBio bioterium-CNPEM

Campinas, February 15th, 2019.

Dr. Rafael Elias Marques
Vice coordinator

Dra. Ângela Saito
Alternate Member
